# Warming Rather Than Increased Precipitation Increases Soil Recalcitrant Organic Carbon in a Semiarid Grassland after 6 Years of Treatments

**DOI:** 10.1371/journal.pone.0053761

**Published:** 2013-01-14

**Authors:** Xiaoqi Zhou, Chengrong Chen, Yanfen Wang, Simeon Smaill, Peter Clinton

**Affiliations:** 1 Graduate University of Chinese Academy of Sciences, Beijing, China; 2 Scion (New Zealand Forest Research Institute), Christchurch, New Zealand; 3 Environmental Futures Centre, Griffith University, Nathan, Australia; DOE Pacific Northwest National Laboratory, United States of America

## Abstract

Improved understanding of changes in soil recalcitrant organic carbon (C) in response to global warming is critical for predicting changes in soil organic C (SOC) storage. Here, we took advantage of a long-term field experiment with increased temperature and precipitation to investigate the effects of warming, increased precipitation and their interactions on SOC fraction in a semiarid Inner Mongolian grassland of northern China since April 2005. We quantified labile SOC, recalcitrant SOC and stable SOC at 0–10 and 10–20 cm depths. Results showed that neither warming nor increased precipitation affected total SOC and stable SOC at either depth. Increased precipitation significantly increased labile SOC at the 0–10 cm depth. Warming decreased labile SOC (P = 0.038) and marginally but significantly increased recalcitrant SOC at the 10–20 cm depth (P = 0.082). In addition, there were significant interactive effects of warming and increased precipitation on labile SOC and recalcitrant SOC at the 0–10 cm depth (both *P*<0.05), indicating that that results from single factor experiments should be treated with caution because of multi-factor interactions. Given that the absolute increase of SOC in the recalcitrant SOC pool was much greater than the decrease in labile SOC, and that the mean residence time of recalcitrant SOC is much greater, our results suggest that soil C storage at 10–20 cm depth may increase with increasing temperature in this semiarid grassland.

## Introduction

The world’s soils contain twice as much as carbon (C) as the atmosphere [1]. As a result, even minor soil organic carbon (SOC) losses from the soils can greatly enhance carbon dioxide concentrations in the atmosphere, which will have a positive feedback on climate [2]. It is predicted that global mean temperature will increase by 1.8–4.0°C at the end of this century [2]. Concurrently with rising temperature, global and regional precipitation regimes are predicted to change as well [2]. Changes in temperature and precipitation can greatly affect C and nitrogen (N) cycling in terrestrial ecosystems [3], [4].

Recently, a meta-analysis including studies from around the world showed that warming had greatly increased soil respiration from the terrestrial ecosystems in the past 5 decades [5]. However, there is much debate whether increases in soil respiration mean that soils are releasing a substantial proportion of their stored C storage into the atmosphere [6]. In fact, the impact of climate change on the C balance of terrestrial ecosystems depends on changes in the plant-derived C entering soils as well as changes in the rate at which SOC is decomposed by heterotrophic organisms and released back to the atmosphere [7]. The SOC can be conceptually divided into several discrete fractions, i.e., labile SOC, recalcitrant SOC and stable SOC, characterized by distinct mean residence times (MRT) from days to years to millenia [7], [8]. Labile SOC accounts for a small fraction of SOC and has a fast turnover with a MRT up to several months. By comparison, recalcitrant SOC is a larger pool and has a slower turnover rate with a MRT from dozens of years to hundreds of years [8]. Therefore, the ability to predict changes in recalcitrant SOC is of more importance than predicting the responses of labile SOC. Until now, studies have documented the decomposition and temperature sensitivity of recalcitrant SOC in response to warming using laboratory incubations [7]–[10] or modeling analysis [11], [12]. However, these studies have focused on only one aspect of the C balance in soils. Available information is inadequate to confidently assess changes in recalcitrant SOC dynamics in response to warming [7]. Acquisition of new data describing recalcitrant SOC responses to warming will greatly advance our knowledge regarding the prediction of soil C storage, which may not be readily evident from gas exchange measurements [3]–[5].

Few studies have examined the effects of increased precipitation/drying on soil respiration in concert with warming [3], [13], not to mention the interactive effects between these treatments [4]. In addition, few studies have examined the effects of increased precipitation on recalcitrant SOC, especially over the long term. Here, we used a long-term, multifactor climate change experiment artificially maintaining increased temperature and precipitation in a semiarid temperate grassland of northern China to investigate the responses of recalcitrant SOC to climate change. We measured labile SOC, recalcitrant SOC and stable SOC fractions at two depths of 0–10 and 10–20 cm in response to warming, increased precipitation and their interactions after 6 years of treatments.

## Materials and Methods

### Ethics Statement

All necessary permits were obtained for the described field studies and we thank Prof. Shiqiang Wan at the Institute of Botany, Chinese Academy of Sciences, for access to the experimental site and assistance in soil sampling.

### Study Site

This study site was established in late April 2005 in a semiarid temperate steppe in Duolun County (42°02′N, 116°17′E, 1324 m a.s.l.), Inner Mongolia, China. The region is characterized as a moderate temperature zone with monsoon climate. Long-term mean annual precipitation and mean annual temperature is approximately 383 mm and 2.1°C, respectively. About 90% of the total precipitation falls during the period from May to October and monthly mean temperature ranges from −17.5°C in January to 18.9°C in July. The soil in this area is classified as chestnut according to Chinese classification or Haplic Calcisols according to the FAO classification with 62.8±0.1% sand, 20.3±0.1% silt, and 16.9±0.1% clay, respectively. Soil bulk density was 1.29±0.04 g cm^−3^ at the 0–10 cm depth and 1.35±0.03 g cm^−3^ at the 10–20 cm depth (Fig. S1). Soil pH was 6.84±0.07. Soil organic C and total N contents were 20.86±3.53 and 1.87±0.31 g kg^−1^, respectively. The plant community at our experimental site was dominated by *Stipa krylovii, Artemisia frigida, Potentilla acaulis, Cleistogenes squarrosa, Allium bidentatum,* and *Agropyron cristatum* [14].

### Experimental Design

This experiment used a paired and nested design with four treatments [4], [14]. There were three blocks with an area of 44×28 m for each block. There was a pair of 10×15 m sub-blocks in each block, in which one plot was assigned as the increased precipitation treatment and the other one as the ambient precipitation treatment. Four 3×4 m plots were established in each 10×15 m sub-block with 1 m distance between the plots. The four plots were randomly assigned to warming and unwarmed control treatments with two replicates. Thus, there were 24 plots in total with six replicates for each treatment [control (C), warming (W), increased precipitation (P), and warming plus increased precipitation (WP)]. There were six sprinklers arranged in two rows in each of the precipitation treatment plot, with each sprinkler covering a circular area with a diameter of 3 m. A total amount of 120 mm precipitation (approximately 30% of the mean annual precipitation at this study site) was applied under the increased precipitation treatment in July and August with approx.15 mm week^−1^. Each warmed plot was heated continuously by a 165×15 cm SR-2420 infrared radiators (Kalglo Electronics, Bethlehem, PA, USA) suspended 2.5 m aboveground since April 28, 2005. One ‘dummy’ heater with the same shape and size as the infrared radiator was used to simulate the shading effect of the infrared radiator in the unwarmed control plot. Long-term monitoring data showed that mean soil temperature at 10 cm depth were 1.39°C and 1.02°C higher in all warming plots than in the control and increased precipitation plots, respectively [15]. Increased precipitation enhanced soil moisture by 1.23% v/v (P<0.01) during the experimental period from 2005 to 2009 as compared to the control plots [14].

### Soil Sampling and Total C and N Measurements

Soil samples were collected from all the 24 plots at two depths of 0–10 and 10–20 cm in early August, 2010. In each plot, one soil core (15 cm in depth and 8 cm in diameter) was taken to get a fresh sample. After passing through a 2 mm sieve, the soil samples were stored at 4°C prior to analysis. The sub-samples were air-dried and stored at room temperature for hot water extraction.

Soil moisture content was determined after being oven-dried at 105°C for 16 hours. Total C (TC) and total N (TN) of soil samples and crop C and N contents were determined using an Isoprime isotope ratio mass spectrometer with a Eurovector elemental analyzer (Isoprime-EuroEA 3000). Soil pH was measured at a 1∶2.5 dry soil/water ratio.

### Measurements of Soil Microbial Biomass C and Labile Organic C

Soil microbial biomass C (MBC) was measured by the chloroform fumigation-extraction method as described before [15]. Briefly, from one soil sample two 10 g (field moist) subsamples were collected and weighed out. One subsample was fumigated with chloroform for 24 h and extracted with 0.5 M K_2_SO_4_ in an end-to-end shaker for 1 h, then the supernatants were filtered through a Whatman no. 42 paper. The other subsample was directly extracted as above. The amounts of total C and N in the fumigated and un-fumigated soil extracts were determined using a SHIMADZU TOC-VCPH/CPN analyzer (fitted with a TN unit).

Cumulative CO_2_-C using laboratory incubation was used to represent labile SOC [8]–[10]. Field soil samples (about 30 g) were adjusted to 60% of the water holding capacity and incubated aerobically in a sealed 1 L jar at 22°C. The CO_2_ evolved from the soils over the 91-day incubation was trapped in 0.1 M NaOH and measured using 0.05 M HCl titration after the precipitation of carbonate with 1 ml of 1 M BaCl_2_. Measurements occurred after 1, 3, 7, 14, 21, 28, 35, 42, 49, 56, 64, 70, 84 and 91 days.

### Measurements of Recalcitrant Organic C and Stable Organic C

The stable SOC was measured using acid hydrolysis method [9]. Water-extractable SOC was removed by filtration through a 0.45 µm glass filter membrane using a pump-vacuum system, then the non-extracted soil material was captured on the membrane. This material was then mixed with a solution of 1 M NaCl to float and remove particulate matter. The remaining soil was then rinsed several times though a fiber membrane with deionized water and then dried at 60°C for 16 hours. After all identifiable fragments of plant material were carefully removed by hand, the soil was acidified in 6 N HCl for 12 h at 116°C to separate acid-soluble and acid-insoluble SOC fractions. After being rinsed with deionized water, the water and soil mixture were passed through a glass fiber membrane to isolate stable SOC. The residue was oven-dried at 60°C for 16 hours, then scraped off the fiber membrane and analyzed to determine the amount of stable SOC on a Eurovector elemental analyzer (Isoprime-EuroEA 3000). The amounts of recalcitrant SOC were determined by subtracting to labile SOC and stable SOC from total SOC for each plot. All SOC fractions were calculated on an area basis at the two depths.
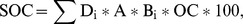
where D_i_, A, B_i_, and OC represent thickness of the soil layer (cm), cross-sectional area (ha), bulk density (g cm^−3^), organic C content (g kg^−1^), respectively; i = 1 and 2. All SOC fractions were expressed as kg C ha^−1^.

### Statistical Analysis

For each soil depth, two-way analysis of variance (ANOVA) for a blocked split-plot design was used to determine the main and interactive effects of warming and increased precipitation on SOC fractions and biochemical properties. A correlation matrix of different properties was based on Pearson’s correlation coefficients (P<0.05). All ANOVA and regression analyses were performed using SPSS 12.0 software (SPSS Inc., USA).

## Results and Discussion

**Figure 1:**
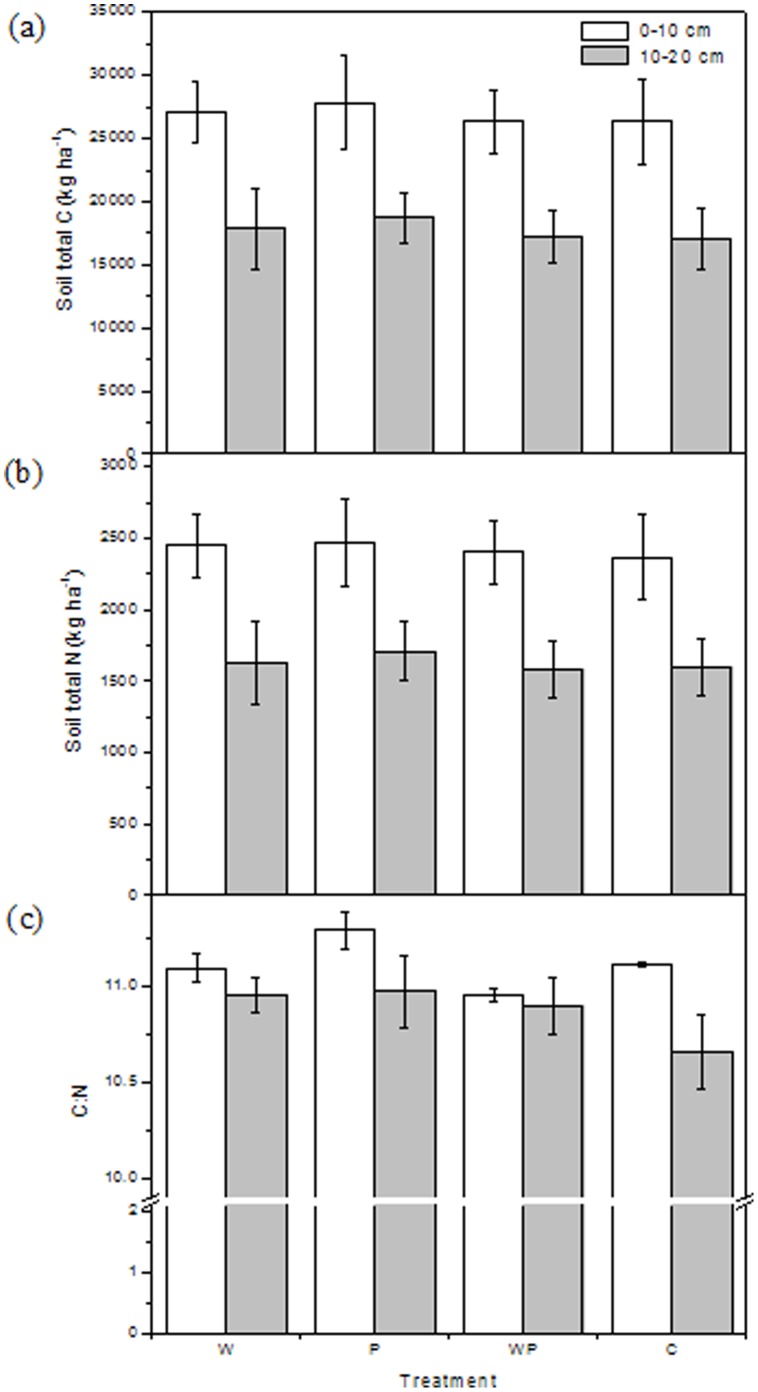
Soil Total C (kg ha ^−**1**^ **; a), N (kg ha** ^−**1**^ **; b) and ratio of C to N (c) (mean ± SE) at two depths under warming, increased precipitation and their interactions.** W, warming; P, increased precipitation; WP, warming plus increased precipitation; C, control.

**Figure 2:**
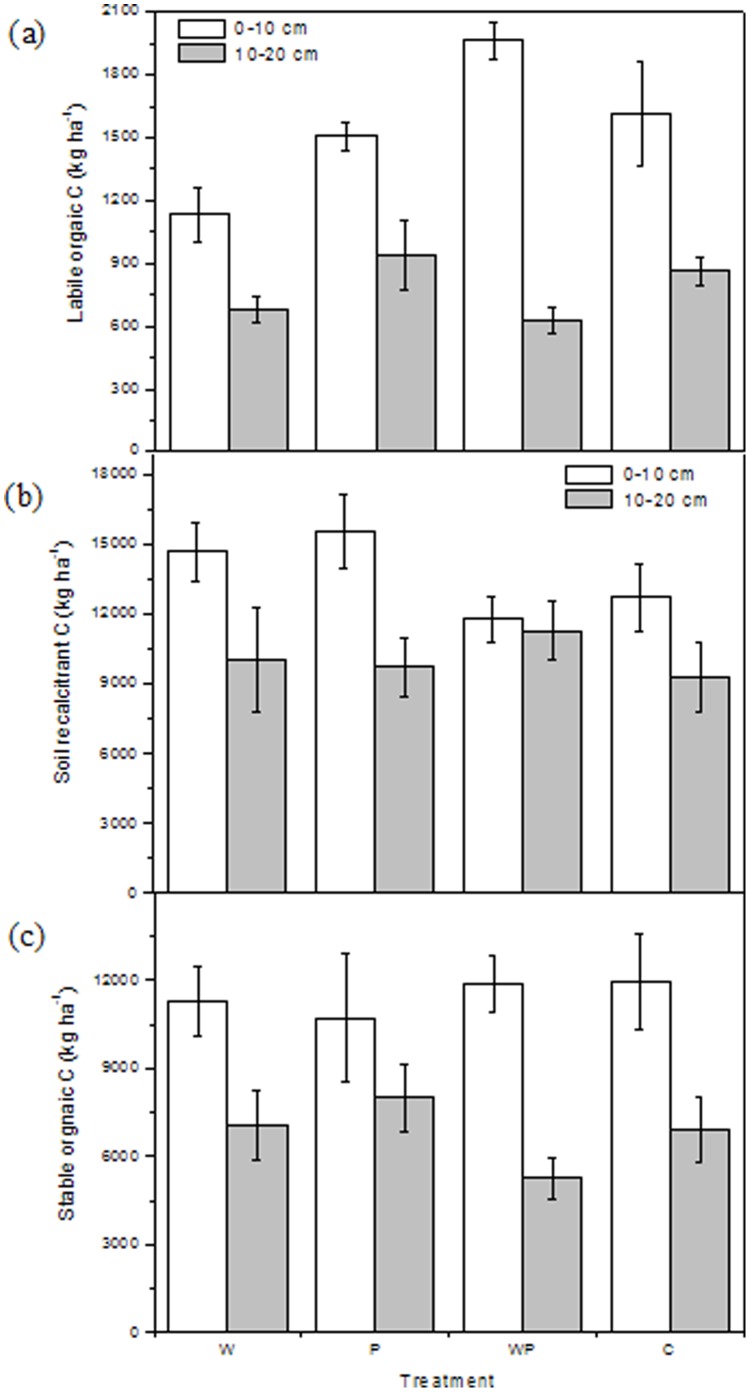
Soil labile organic C (kg ha ^−**1**^ **; a), recalcitrant organic C (kg ha** ^−**1**^ **; b) and stable organic C (kg ha** ^−**1**^ **; c) (mean ± SE) at two depths under warming, increased precipitation and their interactions.** W, warming; P, increased precipitation; WP, warming plus increased precipitation; C, control.

Warming significantly decreased C:N ratios at the 0–10 cm depth (P<0.05) relative to the control, but neither warming nor increased precipitation affected total C and N (Fig. 1) at either depth (Table 1). There were significant interactions between the warming and increased precipitation treatments on the ratios of C to N at the 0–10 cm depth (Table 1). It is very difficult to detect significant changes in soil total C and stable SOC over years as a result of climate change [16], [17]. The lower C:N ratios under warming could be due to faster C losses via soil respiration [4], which was supported by lower labile SOC under warming (Fig. 2a).

**Table 1:** Summary of results (P-value) of two-way factorial _ANOVA_ on the effects of warming and increased precipitation and their interactions on total C, total N, ratio of C to N, microbial biomass C (MBC), labile organic C, recalcitrant organic C and stable organic C at two depths of 0–10 and 10–20 cm relative to control plots^a^.

Treatment	Total C(kg ha^−1^)	Total N(kg ha^−1^)	C:N	MBC(kg ha^−1^)	Labile organicC (kg ha^−1^)^b^	RecalcitrantorganicC (kg ha^−1^)	StableorganicC (kg ha^−1^)
0–10 cm							
W	0.909	0.970	**0.019**	0.535	0.933	0.446	0.886
P	0.909	0.929	0.757	**0.021**	**0.043**	0.902	0.850
W×P	0.709	0.799	**0.033**	0.645	**0.014**	**0.048**	0.574
10–20 cm							
W	0.887	0.847	0.547	0.735	**0.038**	*0.082*	0.146
P	0.844	0.879	0.450	0.736	0.905	0.543	0.754
W×P	0.659	0.731	0.293	0.474	0.539	0.906	0.202

W, warming; P, increased precipitation; WP, warming plus increased precipitation.

a Labile organic C was calculated from cumulative CO_2_-C (mg kg^−1^ dry soil) evolved from 91-day incubation.

P values smaller than 0.05 are bold and *italics* indicates marginal significance (P<0.1).

**Figure 3:**
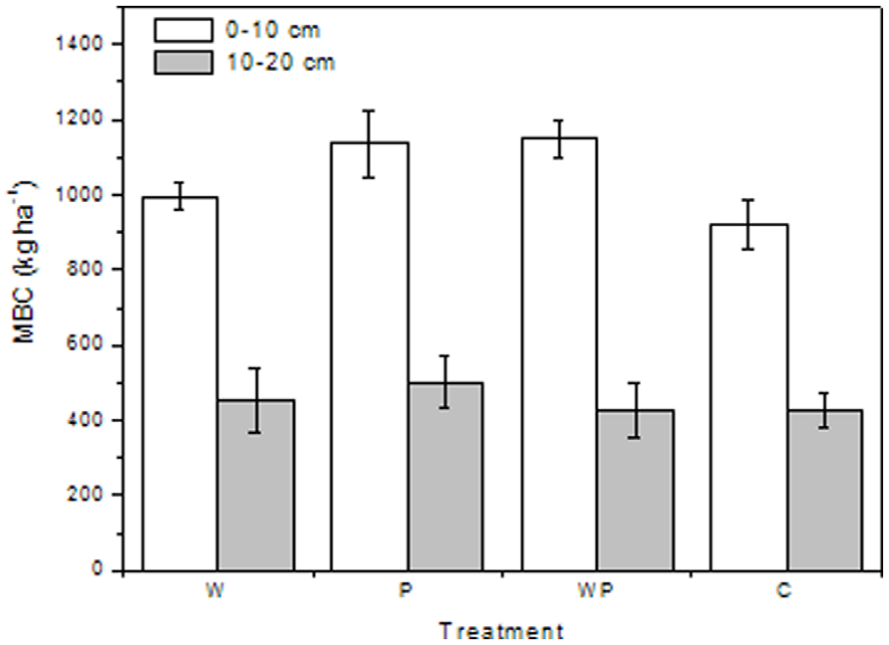
Soil microbial biomass C (MBC; kg ha ^−**1**^ **) at two depths under warming, increased precipitation and their interactions.** W, warming; P, increased precipitation; WP, warming plus increased precipitation; C, control.

As labile SOC is the most active fraction of total SOC, it is sensitive to management practices [18]. Labile SOC can serve as a short term C reservoir and is closely correlated with aboveground biomass [16] and ecosystem respiration [3]. We found that labile SOC accounted for 3.8–7.6% of total SOC across the plots, which agrees with previous research reporting labile SOC as a small fraction of total SOC [19], [20]. The percentage of microbial biomass C in total SOC was 2.5–4.0% and was correlated well with microbial biomass C (r^2^ = 0.71, P<0.001, n = 24) across the treatments. Increased precipitation significantly increased labile SOC at the 0–10 cm depth (Fig. 2a), which was consistent with significantly greater microbial biomass C under increased precipitation (Fig. 3). Greater labile SOC and microbial biomass C under increased precipitation could be due to wetter soils as a result of the increased rainfall treatment, as there were significant correlations moisture contents with labile SOC (r^2^ = 0.72, P<0.001, n = 24) and microbial biomass C (r^2^ = 0.88, P<0.001, n = 24). Our results were supported by previous findings that increased precipitation increased net ecosystem productivity [21], plant biomass [14] and soil respiration [4]. The significant decrease in labile SOC at the 10–20 cm depth with warming was in contrast to a previous study that warming increased soil microbial biomass C and labile organic C in a tallgrass prairie [16]. However, our finding was supported by previous studies in which warming decreased microbial biomass C [22] and soil respiration [3], [4]. The discrepancy between results from our grassland study and the tallgrass prairie study [16] could be due to complexity of ecosystems and various key controlling factors for C cycling in different ecosystems [23].

Neither increased precipitation nor its interaction with warming affected recalcitrant SOC (Fig. 2b) and stable SOC at either depth (Fig. 2c). Acid hydrolysis has been widely used to isolate and quantify stable SOC [24], [25]. No significant changes have been found in stable SOC among the treatments, indicating that stable SOC is insensitive to warming, in contrast to labile SOC [26]. However, warming marginally but significantly increased recalcitrant SOC at the 10–20 cm depth (P = 0.082; Fig. 2b). As recalcitrant SOC contains longer mean residence time compared with labile SOC, this result indicates that there is more C storage (albeit lower labile SOC) with warming compared with ambient temperatures. Warming has been reported to enhance soil respiration based on meta-analysis of gas exchange measurements [5]. However, these findings represented an increase in the rate of C cycling, not meaning that soils are losing a substantial amount of C into the atmosphere [4], [6]. In addition, predictions of climate change largely depend on effects of warming on plant-C input into the soils and understanding of the temperature sensitivity of different SOC fractions [7], [27]. Fang et al. [10] found similar temperature sensitivity for labile SOC and recalcitrant SOC. However, models based on the Arrhenius function show that it is theoretically possible to have increased temperature sensitivity in recalcitrant SOC compared with labile SOC [12], [28], [29], which implies lower recalcitrant SOC with increasing temperature. Many studies have documented one aspect of this process, i.e., temperature sensitivity of SOC decomposition [7], [9], [10]. Given plant-C input, it speculated that plant-derived C could compensate for the accelerated decomposition of recalcitrant SOC in boreal forest soils [30]. However, few studies have examined recalcitrant SOC in response to warming, especially over the long term, as is presented here.

Additionally, warming significantly interacted with increased precipitation in terms of their effects on labile and recalcitrant SOC at the 0–10 cm depth (both P<0.05, Table 1). Compared with the ambient temperature control, warming decreased labile SOC by 31.4% in the ambient precipitation treatment, but warming enhanced it by 24.7% under increased precipitation. Warming increased recalcitrant SOC by 15.6%, but decreased it by 2.1% with increased precipitation at the 0–10 cm depth as compared to the control plots (Fig. 2b). The reason for interaction of warming and increased precipitation is complex. One possible explanation is warming-induced soil drying, as moisture content plays a predominant role in ecosystem community [14], soil respiration [4] and microbial respiration [31] in this semiarid grassland and there was a significant correlation relationship between moisture content and labile SOC. Another explanation for the significant interactions is the potential effects of warming and increased precipitation on root productivity and other plant processes [32], given the close relationships between root turnover and ecosystem C cycling. Increased incidence of extreme rainfall events are a common factor in global warming predictions [2], [33]. However, the responses of recalcitrant SOC processes to concurrent climate warming and changing precipitation are yet to be assessed in this region.

In summary, although warming decreased labile SOC at the 10–20 cm depth, recalcitrant SOC increased by 24.5% at the same depth. Given the longer mean residence time for recalcitrant SOC and the much greater size of this SOC pool, these results suggest that warming may substantially increase soil C storage at the 10–20 cm depth in this semiarid grassland.

## Supporting Information

Figure S1 Soil moisture content (a) and bulk density (b) (mean ± SE) at two soil depths under warming, increased precipitation and their interactions. W, warming; P, increased precipitation; WP, warming plus increased precipitation; and C, control.
(TIF)

## References

[1] Batjes NH (1996) Total carbon and nitrogen in the soils of the world. Eur. J. Soil Sci. 47: 151–163.

[2] IPCC Working Group 1 Climate Change 2007: The physical Science Basis (Cambridge Uni. Press, 2007).

[3] Allison SD, Treseder KK (2008) Warming and drying suppress microbial activity and carbon cycling in boreal forest soils. Glob. Change Biol. 14: 2898–2909.

[4] Liu WX, Zhang Z, Wan SQ (2009) Predominant role of water in regulating soil and microbial respiration and their responses to climate change in a semiarid grassland. Glob. Change Biol. 15: 184–195.

[5] Bond-Lamberty B, Thomson A (2010) Temperature-associated increases in the global soil respiration record. Nature 464: 579–582. 20336143 10.1038/nature08930

[6] Smith P, Fang CM (2010) A warm response by soils. Nature 464: 499–500. 20336128 10.1038/464499a

[7] Davidson EA, Janssens IA (2006) Temperature sensitivity of soil carbon decomposition and feedbacks to climate change. Nature 440: 165–173. 16525463 10.1038/nature04514

[8] Fissore C, Giardina CP, Swanston CW, King GM, Kolka RK (2009) Variable temperature sensitivity of soil organic carbon in North American forests. Glob. Change Biol. 15: 2295–2310.

[9] Conant RT, Drijber RA, Haddix ML, Parton WJ, Paul EA, Plante AF, et al (2008) Sensitivity of organic matter decomposition to warming varies with its quality. Glob. Change Biol. 14: 1–10.

[10] Fang C, Smith P, Moncrieff JB, Smith JU (2005) Similar response of labile and resistant soil organic matter pools to changes in temperature. Nature 433: 57–59. 15635408 10.1038/nature03138

[11] Giardina CP, Ryan MG (2000) Evidence that decomposition rates of organic carbon in mineral soil do not vary with temperature. Nature 404: 858–861. 10786789 10.1038/35009076

[12] Hartley IP, Ineson P (2008) Substrate quality and the temperature sensitivity of soil organic matter decomposition. Soil Biol. Biochem. 40: 1567–1574.

[13] Deng Q, Hui DF, Zhang DQ, Zhou GY, Liu JX, et al (2012) Effects of precipitation increase on soil respiration: a three-year field experiment in subtropical forests in China. PloS One 7: e41493. 22844484 10.1371/journal.pone.0041493 PMC3402392

[14] Yang HJ, Wu MY, Liu WX, Zhang Z, Zhang NL, et al (2011) Community structure and composition in response to climate change in a temperate steppe. Glob. Change Biol. 17: 452–465.

[15] Zhou XQ, Liu X, Rui YC, Chen CR, Wu HW, et al (2011) Symbiotic nitrogen fixation and soil N availability under legume crops in an arid environment. J. Soils Sediments 11: 762–770.

[16] Belay-Tedla A, Zhou XH, Su B, Wan SQ, Luo YQ (2009) Labile, recalcitrant, and microbial carbon and nitrogen pools of a tallgrass prairie soil in the US Great Plains subjected to experimental warming and clipping. Soil Biol. Biochem. 41: 110–116.

[17] Song B, Niu SL, Zhang Z, Yang HJ, Li LH, et al (2012) Light and heavy fractions of soil organic matter in response to climate warming and increased precipitation in a temperate steppe. Plos One 7: e33217. 22479373 10.1371/journal.pone.0033217 PMC3316559

[18] Zhou XQ, Wu HW, Koetz E, Xu ZH, Chen CR (2012) Soil labile carbon and nitrogen pools and microbial metabolic diversity under winter crops in an arid environment. Appl. Soil Ecol. 53: 49–55.

[19] Townsend AR, Vitousek PM, Desmarais DJ, Tharpe A (1997) Soil carbon pool structure and temperature sensitivity inferred using CO_2_ and ^13^CO_2_ incubation fluxes from five Hawaiian soils. Biogeochem. 38: 1–17.

[20] Collins HP, Elliott ET, Paustian K, Bundy LG, Dick WA, et al (2000) Soil carbon pools and fluxes in long-term corn belt agroecosystems. Soil Biol. Biochem. 32: 157–168.

[21] Niu SL, Wu MY, Han Y, Xia JY, Li LH, Wan SQ (2008) Water-mediated responses of ecosystem carbon fluxes to climatic change in a temperate steppe. New Phytol. 177: 209–219. 10.1111/j.1469-8137.2007.02237.x 17944829

[22] Niinistö SM, Silvola J, Kellomäki S (2004) Soil CO_2_ efflux in a boreal pine forest under atmospheric CO_2_ enrichment and air warming. Glob. Change Biol. 10: 1–14.

[23] Allison SD, Wallenstein MD, Bradford MA (2010) Soil-carbon response to warming dependent on microbial physiology. Nature Geosci 3: 336–340.

[24] Fissore C, Giardina CP, Kolka RK, Trettin CC, King GM, Jurgensen MF, et al (2008) Temperature and vegetation effects on soil organic carbon quality along a forested mean annual temperature gradient in North America. Glob. Change Biol. 14: 193–205.

[25] Rovira P, Vallejo VR (2007) Labile, recalcitrant, and inert organic matter in Mediterranean forest soils. Soil Biol. Biochem. 39: 202–215.

[26] Trumbore SE, Chadwick OA, Amundson R (1996) Rapid exchange between soil carbon and atmospheric carbon dioxide driven by temperature change. Science 272: 393–396.

[27] Luo Y (2007) Terrestrial carbon cycle feedback to climate warming. Ann. Rev. Ecol. Evol. Syst. 38: 683–712.

[28] Knorr W, Prentice IG, House JI, Holland EA (2005) Long-term sensitivity of soil carbon turnover to warming. Nature 433: 298–301. 15662420 10.1038/nature03226

[29] Rey A, Jarvis P (2006) Modelling the effect of temperature on carbon mineralization rates across a network of European forest sites (FORCAST). Glob. Change Biol. 12: 1894–1908.

[30] Karhu K, Fritze H, Hämäläinen K, Vanhala P, Jungner H, et al (2010) Temperature sensitivity of soil carbon fractions in boreal forest soil. Ecology 91: 370–376. 20392002 10.1890/09-0478.1

[31] Zhou XQ, Chen CR, Wang YF, Xu ZH, Hu ZY, et al (2012) Effects of warming and increased precipitation on soil carbon mineralization in an Inner Mongolian grassland after 6 years of treatments. Biol. Fert. Soils 48: 859–866.

[32] Bai WM, Wan SQ, Niu SL, Liu WX, Chen QS, Wang QB, et al (2010) Increased temperature and precipitation interact to affect root production, mortality, and turnover in a temperate steppe: implications for ecosystem C cycling. Glob. Change Biol. 16: 1306–1316.

[33] Allan RP, Soden BJ (2008) Atmospheric warming and the amplification of precipitation extremes. Science 321: 1481–1484. 18687921 10.1126/science.1160787

