# Supplementary figures and images for: Warming Rather Than Increased Precipitation Increases Soil Recalcitrant Organic Carbon in a Semiarid Grassland after 6 Years of Treatments
Source: PLoS One. 2013 Jan 14;8(1):e53761. doi: 10.1371/journal.pone.0053761 (PMC3544855; doi:10.1371/journal.pone.0053761)

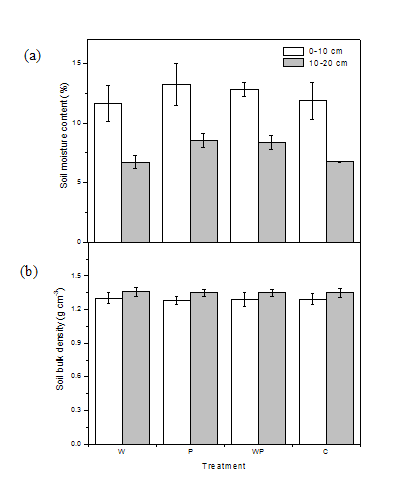

Supplement: Figure S1 — Soil moisture content (a) and bulk density (b) (mean ± SE) at two soil depths under warming, increased precipitation and their interactions. W, warming; P, increased precipitation; WP, warming plus increased precipitation; and C, control. (TIF) [file pone.0053761.s001.tif]
